# Quantitative Proteomic Analysis of MCM3 in ThinPrep Samples of Patients with Cervical Preinvasive Cancer

**DOI:** 10.3390/ijms241310473

**Published:** 2023-06-21

**Authors:** Büşra Köse, Ralf van de Laar, Heleen van Beekhuizen, Folkert van Kemenade, Ahmet Tarik Baykal, Theo Luider, Coşkun Güzel

**Affiliations:** 1 Department of Biochemistry and Molecular Biology, Institute of Health Sciences, Acibadem Mehmet Ali Aydinlar University, 34752 Istanbul, Türkiye; b.kose@erasmusmc.nl; 2 Department of Medical Biochemistry, Faculty of Medicine, Acibadem Mehmet Ali Aydinlar University, 34752 Istanbul, Türkiye; ahmet.baykal@acibadem.edu.tr; 3 Department of Neurology, Erasmus MC, 3015 GD Rotterdam, The Netherlands; t.luider@erasmusmc.nl; 4 Department of Gynecology, Erasmus MC, 3015 GD Rotterdam, The Netherlands; r.vandelaar@erasmusmc.nl (R.v.d.L.); h.vanbeekhuizen@erasmusmc.nl (H.v.B.); 5 Department of Pathology, Erasmus MC, 3015 GD Rotterdam, The Netherlands; f.vankemenade@erasmusmc.nl

**Keywords:** cervical cancer, Parallel Reaction Monitoring (PRM), minichromosome maintenance-3 (MCM3), ThinPrep, protein biomarker, early diagnosis

## Abstract

Triage methods for cervical cancer detection show moderate accuracy and present considerable false-negative and false-positive result rates. A complementary diagnostic parameter could help improve the accuracy of identifying patients who need treatment. A pilot study was performed using a targeted proteomics approach with opportunistic ThinPrep samples obtained from women collected at the hospital’s outpatient clinic to determine the concentration levels of minichromosome maintenance-3 (MCM3) and envoplakin (EVPL) proteins. Forty samples with ‘negative for intraepithelial lesion or malignancy’ (NILM), 21 samples with ‘atypical squamous cells of undetermined significance’ (ASC-US), and 33 samples with ‘low-grade squamous intraepithelial lesion and worse’ (≥LSIL) were analyzed, using cytology and the patients’ histology reports. Highly accurate concordance was obtained for gold-standard-confirmed samples, demonstrating that the MCM3/EVPL ratio can discriminate between non-dysplastic and dysplastic samples. On that account, we propose that MCM3 and EVPL are promising candidate protein biomarkers for population-based cervical cancer screening.

## 1. Introduction

In 2020, according to the International Agency for Research on Cancer report, ~600,000 women were diagnosed with cervical cancer worldwide, and ~350,000 women died because of the disease [1,2]. Cervical cancer is the fourth most common female malignancy worldwide and represents a major global health burden [1]. The main cause of cervical cancer is persistent high-risk Human Papillomavirus (Hr-HPV) infection predominantly acquired via sexual intercourse. Precursors of cervical cancer progress slowly [2], and it may take one to three decades to develop into invasive cancer [3]. Population-based screening programs help to detect precursors of cervical cancer, allowing timely treatment. Currently, an HPV test for Hr-HPV has increasingly been used in population-based screening. At the same time, in regular outpatient clinical settings, cytology is usually combined with HPV screening to increase sensitivity of the test to identify those with a disease and to avoid missing non-HPV-based pathology such as HPV-independent cancers [4,5].

Regarding population-based screening, HPV testing is increasingly replacing cytology and is used for risk assessment followed by triage on the same sample (e.g., genotyping or combination cytology and genotyping) before further clinical referral. This test can be procured with liquid-based cytology (LBC) vials to facilitate so-called immediate triage (on the same sample if needed). In the Netherlands, women are triaged with a combination of genotyping and cytology. Women with an abnormal Pap smear (ASC-US+) in combination with 16- or 18-Hr-HPV positivity are referred for colposcopy. Women with non-16/non-18 Hr-HPV are only referred in case of HSIL+ for directed biopsy. Women with high-risk HPV types other than 16 and 18 are only referred for a directed biopsy in case of HSIL+. Colposcopy-directed biopsy (CDB) consists of a direct visual examination of the cervix with a colposcope, combined with cervical punch biopsy and endocervical curettage. The removed tissue is examined histopathologically (considered the gold standard). Women with high-grade cervical intraepithelial neoplasia (i.e., CIN3+) are offered a loop electrosurgical excision procedure (LEEP) or a large loop excision of the transformation zone (LLETZ). For CIN2+ lesions, a more shared-decision approach can be utilized to avoid overtreatment. A LLETZ procedure has a high accuracy for the diagnosis of CIN, but is an invasive procedure with risk of hemorrhage, stenosis of the cervix, or adverse pregnancy outcomes, among other things [6,7,8].

Unfortunately, current triage tests are still suboptimal [4,9] and a more accurate one-stop test is needed to triage more precisely. Numerous high-throughput omics studies have addressed this issue, but translation of identified molecular markers into clinical practice has not been achieved yet, and these techniques are still ‘research-only’ [10,11,12,13,14].

In a previous study of our group [15], we found that members of the minichromosome maintenance (MCM) protein complex family, especially MCM3, were ~20-fold increased in early- and late-stage squamous cell cervical carcinoma (SCCA) tissue samples compared to adjacent healthy ones. Broadly, MCM proteins have already been known to increase carcinogenesis [16,17]. Since MCMs are cell-cycle proteins, they are naturally not expressed primarily in differentiated healthy somatic cells, whereas uncontrolled dividing cells produce MCMs at high levels [18,19]. This would suggest that MCM3 can be a biomarker to distinguish healthy individuals from cancerous individuals. Against this background, we performed a pilot study using ThinPrep patient samples, focusing on assessing the feasibility of MCM3 protein as a complementary diagnostic tool. The ultimate aim was to develop a biomarker panel that can distinguish the presence of neoplasia among high-risk HPV-positive individuals.

Other methods, such as the p16/Ki67 biomarker test for thin-layer cytology, have shown promising results in triage of Hr-HPV DNA-positive women. However, the potential limitations of testing the p16/Ki67 biomarker include the need for further validation in larger populations and the potential for interobserver variability in interpreting the results [20,21]. The p16/Ki-67 dual-stain assay is a qualitative assay. A complementary diagnostic molecular marker set, such as specific proteins or methylation markers, can enhance the accuracy of population triage programs. Methylation changes associated with HPV infection affect tumor suppressor genes and key signaling pathways (methylation, either viral gene-based or host gene-based). However, limitations in high-throughput methylation assays, including sequencing coverage and stability issues, hinder their translation into clinical practice. Currently, these biomarkers are primarily used in research settings and not in the clinic [10,11,12,13,14,20,22].

A dual biomarker panel was constructed to address sample heterogeneity, including cancerous and healthy cells, and improve reliability. Envoplakin (EVPL) was included in the measurement as a ’healthy epidermal tissue marker’ previously determined [15] as a descending protein alongside the ascending protein MCM3 in SCC tumor tissue. EVPL is a cytoplasmic protein responsible for intermediate filament attachment and maintains cellular junctions, thereby preserving the structural integrity of tissues. It is expressed mainly in squamous epithelia [23,24] and has been found to be downregulated in cervical cancer compared to the healthy [25,26]. Epithelial differentiation proteins such as EVPL are strongly affected by HPV activity [27,28] since the life cycle of HPV is closely linked to these proteins, and HPV infection suppresses epithelial differentiation proteins [28,29,30].

To assess whether MCM3 and EVPL could serve as protein markers for identifying patients with cervix neoplasia, ThinPrep samples from these patients were analyzed with a targeted and untargeted proteomic approach by using a high-resolution tandem mass spectrometry system. Secondly, we evaluated whether these markers could assist in population screening for cervix carcinoma using ThinPrep samples for cytology and HPV detection.

## 2. Results

### 2.1. Defining the Samples

**Figure 1:**
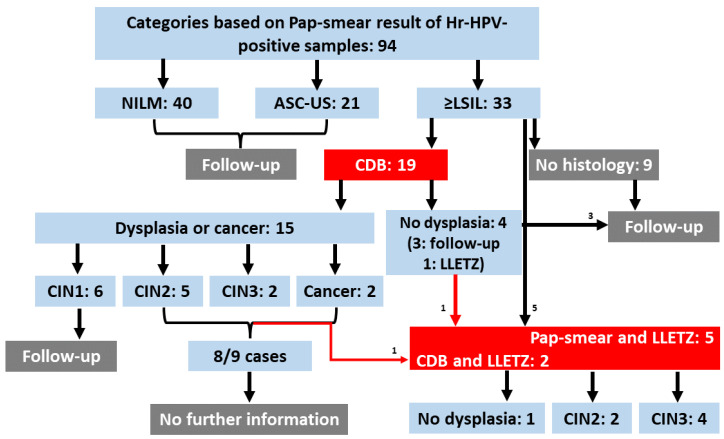
**Flowchart.** Summary of patient information. Ninety-four Hr-HPV-positive samples were collected. Five of them were directly recruited for LLETZ without CDB. Nineteen were recruited to colposcopy. Four colposcopy patients were diagnosed as not-dysplastic; three were released with a 6-month follow-up condition, and the other had suspicious sample material and was sent for LLETZ. Six patients of the colposcopy cohort were diagnosed with CIN1 and were released with a 6-month follow-up condition. Eight cases of nine patients diagnosed with CIN2+ could not be tracked further during the writing process. (CDB = colposcopy-directed biopsy; LLETZ = large loop excision of the transformation zone; NILM: negative for intraepithelial lesion or malignancy; ASC-US: atypical squamous cells of undetermined significance; ≥LSIL: low-grade squamous intraepithelial lesion or worse).

Because of the poor diagnostic power of the current screening methods, sample categorization in these kinds of studies is a great challenge. The collected samples were classified mainly based on cytology scores (NILM, ASC-US, LSIL, HSIL) among the Hr-HPV-positive cases—but also considering the histology results. We preferred using both cytology and histology information because the Pap-smear test has a considerable error probability [31,32,33], and CDB has been found to show discrepancies between the LLETZ results [34,35]. The complete list of clinical information of the cohort is given in the supplementary file (Supplementary File S1) and will be deposited via the PRIDE partner repository [36] with the dataset identifier PXD042918. Briefly, 94 ThinPrep samples were obtained from Hr-HPV-positive patients, with n: 40 NILM, n: 21 ASC-US, and n: 33 ≥LSIL samples, including 7 LLETZ-confirmed samples (Figure 1, Flowchart).

Regarding the total protein concentration of the samples, the distribution of the values was determined as not Gaussian (*p* < 0.0001) according to the Shapiro–Wilk test. In addition, according to the Kruskal–Wallis test, total protein concentration values did not differ significantly (*p* > 0.4) between the groups NILM, ASC-US, and ≥LSIL. In addition, using a simple linear regression analysis method, we estimated the relationship between the variables MCM3 and total protein concentration. The analysis revealed only a weak relationship (correlation value r: 0.02).

To determine the stability of samples, tryptic digestion and Parallel Reaction Monitoring (PRM) measurements of two randomly selected quality control samples (digested ThinPrep) were repeated four times as a technical and methodological repeat. The coefficient of variation (CV%) value was in the range of 6.3–10.3 and 8.9–22.1 for the technical and methodological reproducibility, respectively. We concluded that there was no significant (one-sample Wilcoxon test) instability issue for proteomics analysis within ThinPrep samples after six months’ storage (from the time of collection to the final measurement).

### 2.2. Distribution of MCM3 and EVPL Values over the Groups

**Figure 2:**
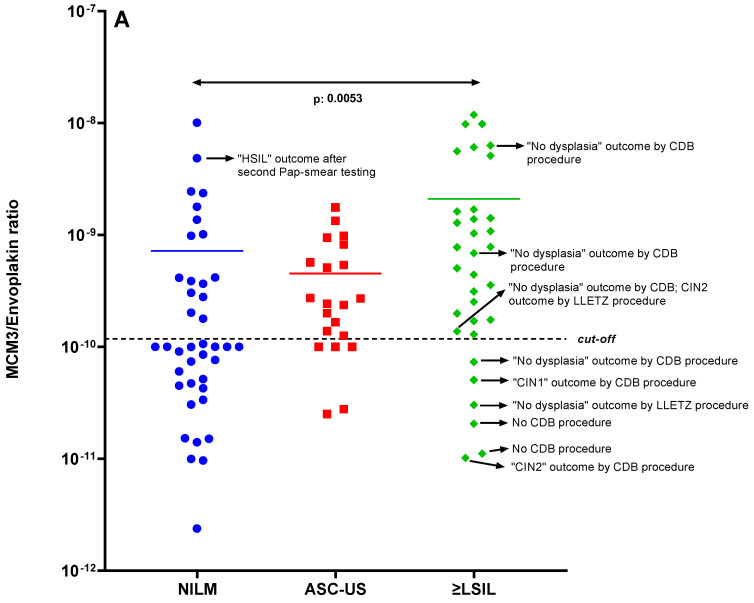

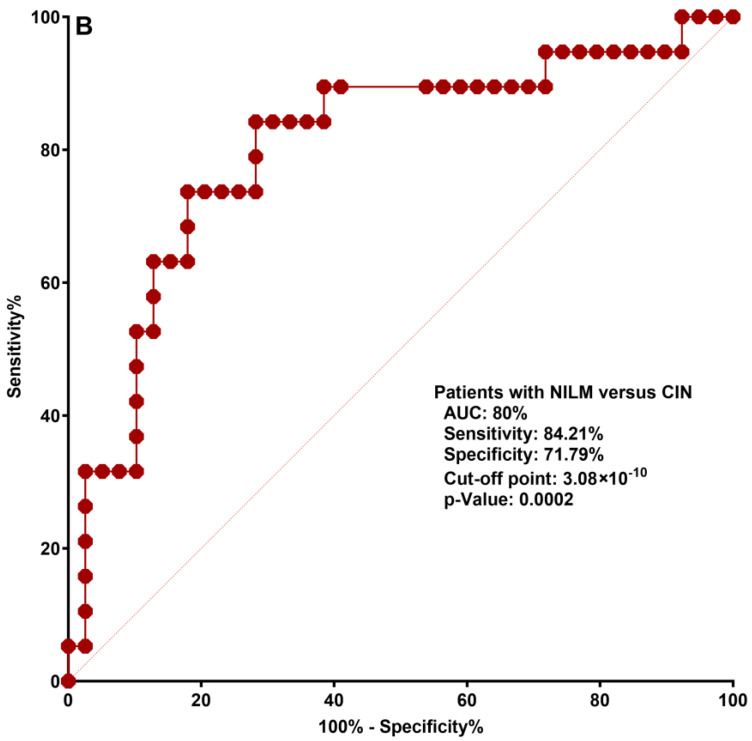

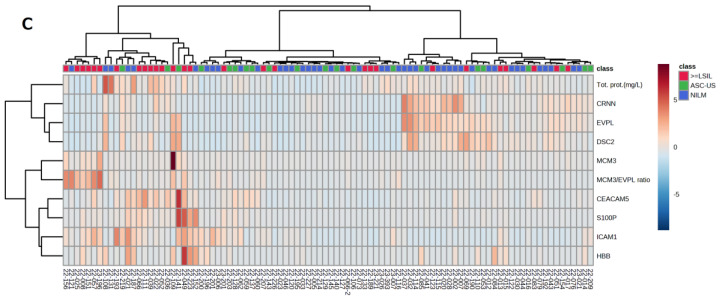
(**A**): Distribution of the MCM3 values of the Hr-HPV-positive samples based on the Pap-smear results. The boxplot for MCM3/EVPL ratio distribution, the mean difference (indicated with a colored horizontal line) between NILM and ≥LSIL groups is significant (*p*: 0.0053). (**B**): Classification analysis: ROC curve. A ROC curve was plotted between NILM (as defined by Pap smear) and dysplastic (CIN1+ as defined by colposcopy) groups and designated as control group (0) and case group (1), respectively. Discrimination power of MCM3 was revealed with AUC of 0.80 with 84.21% sensitivity and 71.79% specificity. (**C**): Unsupervised hierarchical cluster heatmap for targeted protein panel: MCM3, Desmocollin-2, Cornulin, EVPL, S100P, CEACAM5, ICAM1, which were found to be statistically significantly differentially expressed in cervix tissue compared to cervix carcinoma tissue as described in Guzel et al. [15] and the ratios MCM3/Cornulin, MCM3/EVPL, MCM3/Desmocollin-2 proteins. Hemoglobin beta (HBB) was also measured to assess the interference of blood contamination in ThinPrep samples.

PRM results of the ThinPrep samples showed that high-level MCM3 is mainly associated with precancerous stages of the cervix. Figure 2A shows the data distribution of the MCM3/EVPL ratio values over the groups NILM, ASC-US, and ≥LSIL.

The MCM3/EVPL ratio exhibited a significant difference between the NILM and ≥LSIL groups (*p*-value = 0.0053) as determined by Dunn’s multiple comparison test. A cut-off value of 1.178 × 10^−10^ for the MCM3/EVPL ratio between the NILM and ≥LSIL groups was established through ROC curve classification analysis based on cytology scores, yielding an AUC of 0.76, with a sensitivity of 84.38% and specificity of 60.98%. Six samples in the ≥LSIL group remained below the established cut-off value, while 16 surpassed the cut-off point. It is worth noting that some of these samples had CDB diagnoses contrary to the Pap-smear cytology decisions, such as a ≥LSIL Pap-smear report accompanied by a high-level MCM3 value but ‘no dysplasia’ diagnosis by CDB. In order to explore this further, we generated an alternative ROC curve by assigning 39 NILM samples as ‘0’ and classifying 17 dysplastic samples and 2 cancer samples as ‘1’, as determined by histology (CIN1+) and cytology (NILM). According to this ROC curve, MCM3/EVPL has an improved 0.80 AUC with 84.21% sensitivity and 71.79% specificity (Figure 2B).

To further evaluate the relationship of the dependent variables measured in PRM data, we plotted an unsupervised hierarchical cluster heatmap from the same perspective (Figure 2C). This heatmap, based on a dataset including samples from NILM, ASC-US, and ≥LSIL groups, displays two main clusters: cancer markers (MCM3, CEACAM5, S100P, ICAM1) on the left and healthy markers (CRNN, EVPL, DSC2) on the right side of the figure. Samples in the ≥LSIL group predominantly cluster in the left part of the figure, whereas the NILM samples mainly cluster on the right side. No correlation between the total protein and HBB concentration with MCM3 and the MCM/EVP ratio suggests that high MCM3 and MCM3/EVPL is not due to the influence of blood contamination or high protein levels. The abundance levels of proteins are indicated with red (high) and light blue (low, zero level).

### 2.3. Evaluation of Concordance between LLETZ Procedure Diagnosis and MCM3 and Envoplakin Proteins

**Figure 3:**
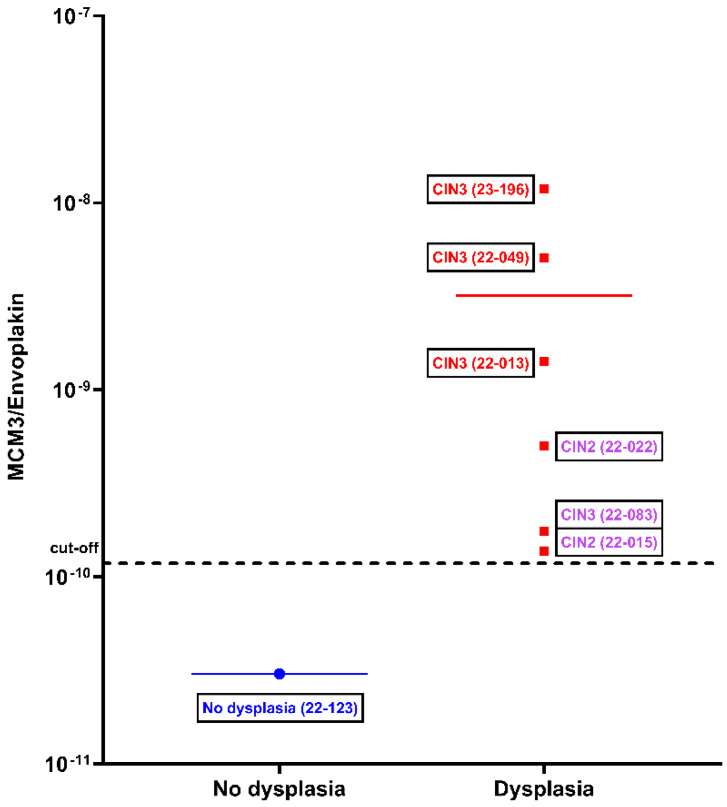
MCM3/EVPL ratio values of the LLETZ patients. On the left, one patient sample with ‘no dysplasia’; on the right, six patient samples with CIN2 or CIN3 diagnosed positively by the LLETZ procedure. Three samples indicated in purple belong to patients who underwent a LLETZ procedure twice because of the recurrence of HPV-associated cervical malignancy after the first occasion. The mean difference is indicated with a colored horizontal line.

A LLETZ procedure covers the surgical excision of the transformation zone on the cervix by a loop to prevent tumor development for patients with CDB-confirmed CIN2+ diagnosis [37,38]. This technique removes a relatively large tissue part from the cervix and is considered the gold standard with utmost diagnostic accuracy [35,39,40] through histopathological analysis of the removed tissue. Considering this, we plotted the samples from the patients selected for the LLETZ procedure separately. Nonetheless, the LLETZ procedure is not often performed [37], as it is a surgical treatment, not a routine diagnostic approach. Therefore, only 7 out of 94 HPV-positive patients had undergone the LLETZ procedure, and the samples of these patients served to validate the discrimination of MCM3/EVPL as confirmed with the most accurate gold standard. The CIN3 samples located on the right top of the graph in Figure 3 have a 40–60-fold higher MCM3/EVPL ratio value compared to the value of the non-dysplastic sample on the left. The clustered three samples from three patients who had undergone a LLETZ procedure twice are also located above the MCM3/EVPL ratio cut-off.

## 3. Discussion

We showed that ThinPrep samples can be used to measure protein markers for cervical malignancies. The MCM3 protein was significantly upregulated (*p*: 0.0053) in the cervical dysplasia group (≥LSIL) compared to the control (NILM) group. An epithelial protein normally present in the cervix (EVPL) was used to compensate variations in the protein content obtained in ThinPrep samples.

Shotgun proteomic analysis identified several hundred proteins per ThinPrep LBC specimen, and previously described differentially present cervix carcinoma proteins were individually quantified within each patient sample using the PRM method (see Supplementary File S2 for a detailed list of peptide targets). We preferred using the MCM3/EVPL ratio value since these two proteins were most discriminant to separate case and control. The increase in MCM3 shows the velocity of the DNA replication and correlates strongly with cell division. In contrast, the decrease in EVPL shows that the cells have started to lose their epithelial features and attachment to the basal membrane, which is a step for epithelial-mesenchymal transition, cellular migration, and invasion. Thus, EVPL aids the improvement of MCM3 to discriminate the neoplastic samples among the Hr-HPV-positive samples. The heatmap figure in Figure 2C also suggests that MCM3 is one of the most important variables in explaining the differences between the samples, and EVPL correlated negatively with MCM3. The MCM3/EVPL ratio value significantly influenced the separation of the samples in the plot.

In our previous study, the difference (*p* < 0.0001) in MCM3 levels between the cells separated by laser capture microdissection (LCM) from SCC tumor tissue and adjusted healthy tissue was 20-fold and 4-fold, respectively [15]. Unsurprisingly, in this study, the difference (*p*: 0.0053) between the NILM and ≥LSIL groups was smaller since most of these patients did not have a cervical tumor but rather a lesion or dysplasia, and cells were not enriched by capturing and separating by LCM or any other technique. Nonetheless, we could discriminate the Pap scores (NILM versus ≥LSIL) among the 94 Hr-HPV-positive individuals. Notably, these outcomes ought to be interpreted from the perspective that the Pap-smear test has poor sensitivity (47%) and specificity (64%) ratios [4,9,33,41,42,43]. In this regard, according to Figure 2B, we can conclude that MCM3/EVPL has considerably improved the sensitivity (from 47% to 84.21%) and specificity (from 64% to 71.79%) of the overall screening; the sensitivity and specificity could probably be higher if the diagnostic information were accurate.

In Figure 2A, the boxplot showed the distribution of the ratio values of MCM3 and EVPL proteins among Hr-HPV positive individuals, which were grouped based on NILM, ASC-US, or ≥LSIL smear reports. Since undetermined significance is an unspecified condition, we separated the patient group with ASC-US score as a third group in the middle of the control and case groups [44,45]. Altogether, the uncertainty in sample classification is based on the current techniques used. As an example of the inherent uncertainty of the Pap-smear test, a Pap-smear-tested patient received a NILM cytology outcome during the initial gynecology visit. However, the result of a new Pap-smear test four months later was a reason to change this cytology outcome to HSIL (Figure 2A on the left, with a high-level MCM3 originally in agreement with HSIL).

The six case samples pointed out on the right side of the graph in Figure 2A, which are below the cut-off value, can be considered false positives. Firstly, the samples of two patients with abnormal Pap-smear cytology reports, who had been diagnosed with ’no dysplasia’ by CDB and LLETZ should be considered control samples rather than case samples. Two patients were diagnosed with CIN1 and CIN2, respectively, by CBD. However, the CDB method has been reported to have only a 60% consistency with the LLETZ histopathology result [34,35,40,46]. Additionally, despite abnormal cytology reports, two patients were not recruited for CDB (annotated as ‘no CDB procedure’).

To overcome these discrepancies and improve the power of the alignment with the gold standard in the ROC curve, we used the histology information for the case group because CDB is more reliable than the Pap smear [5,43]. NILM (with no histology information) and dysplasia (with CIN1+ histology information by CDB and/or LLETZ) were used as 0–1 assignments to determine the clinical group discrimination power shown in Figure 2B. The resulting ROC curve showed a powerful classification.

In Figure 3, with seven ThinPrep samples of LLETZ patients following an abnormal Pap-smear and/or CDB result, an LLETZ-confirmed graph was plotted based on MCM3/EVPL ratio values. It should be emphasized that the ‘no dysplasia’ sample on the left side of the graph is an unexpected LLETZ result demonstrating the limits of the Pap-smear specificity.

Nevertheless, successful discrimination can be seen in Figure 3; the non-dysplastic sample is located at the bottom of the graph with a lower value, while three CIN3 samples are located at the top of the graph, suggesting that the discrimination of dysplastic and non-dysplastic samples with the MCM3/EVPL ratio is in accordance with the gold-standard method LLETZ. Notably, the purple samples in the graph represent a different sample class consisting of three secondary LLETZ patients. These samples belong to ‘surgical follow-up cervix carcinoma patients’.

ThinPrep samples are easy to obtain and can be easily integrated into various tests for several types of biomolecules. The samples can be stored at room temperature for at least one year.

A few proteomics studies focused on cervical pre-cancer biomarkers in ThinPrep samples. Gu et al. [47] described potential protein markers in ThinPrep cervical cytological specimens. They used the linear ion trap coupled with a Fourier-transform mass spectrometer. More than 1000 unique proteins were identified from nine normal and nine dysplastic samples. More than 200 proteins were found to be expressed with a 3-fold difference.

Zappacosta et al. [48] conducted a gel-based proteomics analysis. The analysis revealed proteins that are constitutively present in healthy cervical samples and strictly modulated during carcinogenesis. Testing the optimum storage duration of the ThinPrep samples revealed that protein recovery decreases while storage duration increases. In this context, we also repetitively measured—through repetitive PRM assays at different time points—three randomly selected samples from our cohort, and the results certify that the protein concentration of the samples remained stable throughout six months.

A limitation of this study and similar studies is the general issue associated with the uncertainty of the Pap-smear and CDB techniques, resulting in inherent inaccuracy [34,46,49,50,51,52] and discrepancy [34,49]. Therefore, the sample classification and, subsequently, statistical analysis create dilemmas regarding the alignment of a new target for clinical diagnosis.

The proposed test has the potential to be a regulatory test in population screening because it is relatively rapid, robust, and adaptable to the clinical setting because of the traditional sampling method used. Seven samples of patients who underwent the LLETZ procedure showed that the MCM3/EVPL ratio is able to discriminate the non-dysplastic and precancerous samples in the present study. We hypothesize that this method offers a complementary triage tool for supporting the current screening methods. Conducting additional validation is needed for these two candidate biomarkers in an external population-based screening cohort.

## 4. Material and Methods

### 4.1. Sample Collection and Classification

The sample collection was carried out following the current ThinPrep sampling guidelines [53] in the Netherlands, and all patients provided written informed consent. The study was approved by the Medical Ethics Review Board of the Erasmus University Medical Center, Rotterdam, The Netherlands (protocol reference number MEC-2022-0314). The current norm of Good Clinical Practice corresponds to the standards established by the “2013 Helsinki Declaration”. To administer confidentiality standards, a random code was assigned to each patient sample to anonymize it.

The ThinPrep cell fixation solution, which contains 35–55% methanol, serves as a long-term stabilizer for biological material [40,46,47,54]. The samples were stored at room temperature after collection. These ThinPrep smear samples were obtained between August 2022 and January 2023 at the Department of Gynaecology, Erasmus University Medical Center. They were primarily collected during routine check-up following abnormal cytology or histology reports, surgical treatment, or in cases where Pap-smear testing was indicated, such as abnormal or postmenopausal bleeding. As a result, these samples are considered opportunistic patient samples rather than population-screening-based samples. After pathology evaluation by an expert pathologist (FvK) for patient diagnosis, the samples were sent to the Erasmus MC Clinical and Cancer Proteomics Laboratory for mass spectrometry analysis.

Since the sample material was a cytological scraping specimen collected for cytological evaluation, some patients were released following a negative for intraepithelial lesion or malignancy (NILM) or atypical squamous cells of undetermined significance (ASC-US) diagnosis report. Therefore, a minority of the patients who were diagnosed with a low or high-grade squamous intraepithelial lesion or worse (≥LSIL: LSIL, HSIL, or carcinoma) also had a colposcopy with biopsy and CIN-stage diagnosis (see Supplementary File S1).

In this pilot study, a total of 189 samples were collected to assess the clinical discriminative power of MCM3 and EVPL. The average age of the patients was 43. All samples were blindly and chronologically measured over a span of five months. Initially, the samples were collected prospectively for diagnostic purposes, and any remaining samples were stored for three months to address possible additional diagnostic queries. Subsequently, these stored samples were utilized for this retrospective study. However, HPV-negative samples (n = 95) were excluded from the targeted analysis but were still subjected to DDA (Data-Dependent Acquisition) shotgun analysis and made accessible through the PRIDE repository [36].

### 4.2. Total Protein Quantification

The total protein concentration of the samples was determined using a Pierce BCA™ Protein Assay Kit (Thermo Fisher, Waltham, MA, USA, #23238) bicinchoninic acid (BCA) protein assay. A hundred microliters (µL) volume of each sample was fully homogenized by vortexing and placed into LoBind^®^ 1.5 mL tubes (Eppendorf, Hamburg, Germany, # 0030108116), and 10 µL 0.1% RapiGest surfactant (Waters™ #186008090) was added and strongly sonicated for 30 s on a horn sonifier bath (Ultrasonic Disruptor Sonifier II, Bransons Ultrasonics, Danbury, CT, USA) at 85% amplitude to disrupt the tissue particles. To homogenize the tissue fragments, the mixture was incubated at 60 °C for 10 min by shaking at 400 rpm. Finally, samples were centrifuged (in an Eppendorf centrifuge tube) at 14,000 rpm for 10 min at +4 °C to pellet any particles. Notably, the whole sample was taken and spun before the spectrophotometric measurement.

The obtained clear material of the patient samples was added to the well plate with blank and bovine serum albumin (BSA) standards which were serially diluted with the blank matrix (ThinPrep fixative). A blank, just ThinPrep fixative, was taken and placed in the plate as a baseline value of the assay. BCA containing working reagent was added in all wells, incubated at 37 °C for 30 min, and absorbance values were measured at 562 nm.

### 4.3. Sample Preparation

For tryptic digestion, 1 mL of each fully homogenized ThinPrep sample was directly taken into 1.5 mL Eppendorf tubes and centrifuged at 8000 rpm, for 10 min, at +4 °C. The supernatant of samples containing the methanol fixation buffer was removed. The pellet was collected, and 100 µL 0.1% RapiGest surfactant (Waters, Milford, MA, USA) dissolved in 50 mM ammonium bicarbonate was added and then strongly sonicated for 30 s on a horn sonifier bath (Ultrasonic Disruptor Sonifier II, Bransons Ultrasonics, Danbury, CT, USA) at 85% amplitude and subsequently incubated at 95 °C for 5 min by shaking at 450 rpm to homogenize any tissue fragments. For reduction, alkylation, and quenching of the proteins, 1 µL 0.5 M dithiothreitol (DTT), 3 µL 0.5 M iodoacetamide (IAA), and again DTT (2 µL 0.5 M) were used, respectively. Tryptic digestion was performed with 10 µL 100 ng/µL trypsin (Promega Trypsin Gold, Mass Spectrometry grade, 100 μg/mL in 3 mM Tris-HCl, pH 8.8) and overnight incubated at 37°C. Digestion was ended with trifluoroacetic acid to reach pH < 2, and samples were centrifuged at 14,000 rpm at 4 °C for 30 min. The clear supernatant was collected, and 100 µL of each sample was transferred into LC glass vials (Thermo Fisher Scientific, Waltham, MA, USA).

### 4.4. Proteomics Analysis: Device Conditions, Data Collection, and Identification

A 0.5 µL volume injection of each digested ThinPrep sample was subjected to separation using an Ultimate 3000 HPLC system (Thermo Fisher Scientific, Waltham, MA, USA). Subsequently, the samples were analyzed using Orbitrap Lumos, Fusion, and Exploris 120 mass spectrometers (Thermo Fisher Scientific, Waltham, MA, USA) operating in DDA mode to obtain a general overview of protein abundances and identify potential outliers. For targeted analysis of the protein panel consisting of MCM3, Desmocollin-2, Cornulin, EVPL, S100P, CEACAM5, and ICAM1, the PRM targeted mode was employed. The Fusion system was used for DDA measurements, while the Exploris system was used for PRM measurements. For the Orbitrap Lumos and Fusion DDA measurements, samples were loaded onto a trap column (PepMap C18, 300 μm ID, 5 mm length, 5 μm particle size, 100 Å pore size; Thermo Fisher Scientific, Waltham, MA, USA), for 10 min using 0.1% trifluoroacetic acid as loading solvent at a flow rate of 20 μL/min. The trap column was switched in line with the analytical column (PepMap C18, 75 μm ID × 250 mm, 2 μm particle size and 100 Å pore size, Thermo Fisher Scientific). Peptides were eluted with a 30 min acetonitrile gradient ranging from 3% to 30% (and formic acid concentration from 0.1% to 0.08%, respectively) at 250 nL/min. For electrospray ionization, nano ESI emitters (New Objective, Woburn, MA, USA) were used with an applied spray voltage of 1.8 kV. A DDA method was used with a survey scan from 375 to 1550 Th at 120,000 resolution (AGC target 400,000) and consecutively isolated and fragmented by Higher-energy C-trap dissociation (HCD) at fixed 30% collision energy (AGC target 10,000) of the most abundant precursors in the linear ion trap until a duty cycle time of 3 s was reached (‘Top Speed’ method). For the MS/MS analysis, precursor masses were selected and subsequently excluded from further fragmentation for 60 s to minimize redundancy in the data. The proteins in the samples were identified by exporting features with recorded MS/MS spectra using ProteoWizard software (version 3.0.9248). The resulting .mgf files were then submitted to Mascot (version 2.3.01, Matrix Science) for protein identification against the human UniProtKB/Swiss-Prot database (version 2012_12, Human Taxonomy, 20,395 entries) using trypsin digestion and fragment ion mass tolerance of 0.5 Da, parent ion mass tolerance of 10 ppm, and a maximum number of missed cleavages of 2. Oxidation of methionine was specified as a variable modification, and carbamidomethylation of cysteine as a fixed modification. The resulting peptide data from the Mascot search were processed using Scaffold (version 5.3.0, Proteome Software Inc., Portland, OR, USA) for summarization and filtering. The number of proteins was determined based on the peptide data according to the criteria of the ‘Peptide Prophet’ algorithm, with a probability of >95% and protein identification with a probability of >99%, and at least one peptide identification was allowed.

The DDA data will be deposited to the ProteomeXchange Consortium (http://proteomecentral.proteomexchange.org, accessed on 15 May 2023) via the PRIDE partner repository [36] with the dataset identifier PXD042919.

### 4.5. Parallel Reaction Monitoring Quantification of MCM3 Referenced via Stable Isotope Labeled (SIL) Peptides

The PRM method was used to quantitatively measure the levels of specific peptides (see list peptide targets in Supplementary File S2) in ThinPrep digests. The data were analyzed using Skyline-daily software (version 22.2.1.501, McCoss Lab, University of Washington, Seattle, WA, USA). The analytical parameters for the PRM Orbitrap Lumos measurements were applied similarly to the DDA measurements mentioned earlier, with some alterations made. We used the targeted MS/MS mode, with a lock mass of Fluoranthene (202.0777 Da), to ensure accurate measurement of the peptides. The targeted MS/MS mode was set up with the following parameters: isolation width of 0.7 Da, HCD fragmentation with an HCD collision energy of 25–28 depending on the peptide target, dynamic ion injection time mode (by setting the AGC target to standard mode), and an Orbitrap resolution of 60,000. Precursor ion selection was non-scheduled, and each duty cycle incorporated both endogenous and stable isotope-labeled targeted MS/MS scans.

For Orbitrap Exploris measurements in the PRM mode, samples were loaded onto a trap column (PepMap C18, 300 μm ID, 5 mm length, 5 μm particle size, 100 Å pore size; Thermo Fisher Scientific), for 4.5 min using 0.1% trifluoroacetic acid as loading solvent at a flow rate of 20 μL/min. The trap column was switched in line with the analytical column (PepMap C18, 75 μm ID × 150 mm, 3 μm particle size, and 100 Å pore size, Thermo Fisher Scientific). Peptides were eluted with a 30 min acetonitrile gradient ranging from 3% to 32% (and formic acid concentration from 0.1% to 0.08%, respectively) at a flow rate of 1.25 µL/min. All LC solvents were purchased at Biosolve (Valkenswaard, The Netherlands). Eluting peptides were measured online at 214 nm in a 3 nL nano flow cell (Thermo Fisher Scientific).

In order to evaluate the expression levels between the precancerous stages, the peptides of eight proteins were included in the PRM target list (Supplementary File S2). From the targeted proteins in the current study, which were differentially found in our previous tissue-based cervical carcinoma study [15] the MCM3, CEACAM5, ICAM1, and S100P proteins were designated as ‘cancer markers’ (increased in cervical cancer compared to healthy tissue). On the other hand, Cornulin (CRNN), Desmocollin-2 (DSC2), and Envoplakin (EVPL) proteins were designated as ‘healthy markers’ that are expected to be declining in the patient group compared to the control group. In addition, we measured Hemoglobin beta to determine if blood contamination has any effect on the PRM data from the ThinPrep samples.

The highest signal peptides of MCM3, CEACAM5, ICAM1, and S100P were integrated using Skyline-daily software (version 22.2.1.501, McCoss Lab, University of Washington, Seattle, WA, USA). The concentration was then calculated using the peak area ratio between endogenous and the SIL peptides. On the other hand, for CRNN, DSC2, EVPL, and HBB, a minimum of five y-transitions were considered by using the total peak areas of endogenous peaks.

The PRM data will be deposited to the ProteomeXchange Consortium (http://proteomecentral.proteomexchange.org, accessed on 15 May 2023) via the PRIDE partner repository [36] with the dataset identifier PXD042918s.

### 4.6. Stability of the Protein Content of the Samples

In this study, measurements were conducted on a monthly basis using a sample set comprising an average of 40 samples. These samples had been stored at room temperature for three months to allow for potential clinical follow-up questions, with the collection period spanning from December 2022 to March 2023. Each sample set was subjected to LC-MS/MS analysis without specific selection criteria. The objective was to assess variations between injections, different mass spectrometry systems, and the stability of the sample material. To evaluate the stability of the samples, two randomly selected quality control samples (digested ThinPrep) underwent tryptic digestion and PRM measurement. This process was repeated four times as a technical and methodological replication. It is important to note that the time zero point for the analysis was set as the third month following sample collection, as the samples arrived at the laboratory after being stored for three months.

### 4.7. Statistical Analysis

The non-parametric mean difference Kruskal–Wallis test and Dunn’s multiple comparison tests were used to evaluate differences between independent variables with *p*-value calculation; receiver operating characteristic (ROC) curve analysis revealed the MCM3/EVPL ratio cut-off value; and correlation analysis revealed the relationship between the different parameters. All tests were conducted, and figures were generated using SPSS (IBM24 for Windows, Inc., Chicago, IL, USA) and GraphPad Prism (version 9.5.1 for Windows, San Diego, CA, USA) software. An unsupervised hierarchical cluster heatmap analysis was performed to assess the relationship between the dependent variables measured in the PRM data. The analysis was carried out using the freely available MetaboAnalyst platform (https://www.metaboanalyst.ca) (accessed on 15 May 2023) [55].

## 5. Conclusions

A biobank was established at Erasmus University Medical Center Rotterdam, consisting of ThinPrep samples with information about clinicopathological characteristics from women who volunteered and had cervical complaints. A targeted PRM method was successfully employed using samples from this biobank for protein analysis using a high-throughput tandem MS system. Our analysis, in agreement with the literature, considered that false-negative and false-positive results are inevitable in cervical precancer screening due to significant misdiagnosis associated with current methods (colposcopy, cytology, histology).We investigated the candidate protein biomarkers MCM3 and EVPL to enhance routine cervical screening. By incorporating histology data (CIN1+) alongside cytology findings of NILM, we observed an improved sensitivity of 84.21% and specificity of 71.79% versus a sensitivity of 84.38% and specificity of 60.98% for cytology only. Notably, the MCM3/EVPL ratio demonstrated 100% successful agreement when compared to LLETZ-histology confirmed samples; however, it is important to note that the sample size (n = 7) was limited due to the practical implications of applying LLETZ.

Despite the positive results with MCM3 and EVPL, the evaluation relies on the imperfect current screening methods. Our pilot study suggests that further research with more extensive, well-characterized samples is needed to develop a supplementary cervical cancer test that enhances current practices and improves treatment for cervical cancer in opportunistic and population-based patients.

## Data Availability

The data presented in this study are available on request from the corresponding author and will be uploaded in a publicly available repository if the manuscript is accepted.

## Supplementary Materials

The following supporting information can be downloaded at: https://www.mdpi.com/article/10.3390/ijms241310473/s1.

## Abbreviations

ASC-US	Atypical squamous cells of undetermined significance
CDB	Colposcopy-directed biopsy
CIN	Cervical intraepithelial neoplasia
EVPL	Envoplakin
HPV	Human Papillomavirus
Hr	High-risk
HSIL	High-grade squamous intraepithelial lesion
LLETZ	Large loop excision of the transformation zone
LSIL	Low-grade squamous intraepithelial lesion
≥LSIL	LSIL and the other higher abnormalities
MCM3	Minichromosome maintenance-3
NILM	Negative for intraepithelial lesion or malignancy
Pap smear	Pappanicolau stain cellular smear test
PRM	Parallel Reaction Monitoring
SCC	Squamous cell carcinoma
SIL	Squamous intraepithelial lesions
