# Supplementary material for: Quantitative Proteomic Analysis of MCM3 in ThinPrep Samples of Patients with Cervical Preinvasive Cancer
Source: Int J Mol Sci. 2023 Jun 21;24(13):10473. doi: 10.3390/ijms241310473 (PMC10342024; doi:10.3390/ijms241310473)
Supplement: Supplementary file 1 [file ijms-24-10473-s001.zip › 20230516_Supplementary File S2 PRM Peptide List.pdf]

Supplementary_File_PRM_Peptide_List

The parameters used in a Parallel Reaction Monitoring (PRM) mass spectrometry assay to detect and quantify various peptides (endogenous and stable-isotope labeled) from different proteins. The name denotes these proteins before the ">" symbol, and the peptide sequence is specified after the ">" symbol. The presence of a "#" before the charge state refers to a labeled form of the peptide used as an internal standard for the PRM assay.

Compound	m/z	z	t start (min)	t stop (min)	HCD Collision Energy (%)	Scan Range (m/z)
CEACAM5>SDLVNEEATGQFR#2+	738.3537	2	0	32	25.5	150-1500
CEACAM5>SDLVNEEATGQFR2+	733.3495	2	0	32	25.5	150-1500
CEACAM5>TLTLLSVTR#2+	507.3151	2	0	32	27	150-1000
CEACAM5>TLTLLSVTR2+	502.3109	2	0	32	27	150-1000
CRNN>ISPQIQLSGQTEQTQK2+	893.4707	2	0	32	25	150-1700
CRNN>ELYSYLR2+	472.2478	2	0	32	25	250-1000
CRNN>QEWSSTHPR2+	564.2651	2	0	32	25	250-1200
DSC2>GPGVDQEPR2+	477.7356	2	0	32	25	250-1100
DSC2>YTYSEWHSFTQPR3+	567.9267	3	0	32	25	250-1800
EVPL>AATQELALLISR2+	643.3774	2	0	32	25	250-1400
EVPL>LSGSLDEEVGR2+	581.2909	2	0	32	25	250-1300
HBB>SAVTALWGK2+	466.7636	2	0	32	25	250-1000
HBB>LLVVYPWTQR2+	637.8664	2	0	32	25	250-1350
ICAM1>ASVSVTAEDEGTQR#2+	730.3486	2	0	32	25.5	150-1500
ICAM1>ASVSVTAEDEGTQR2+	725.3444	2	0	32	25.5	150-1500
ICAM1>DGTFPLPIGESVTVTR#2+	849.9505	2	0	32	29	150-1700
ICAM1>DGTFPLPIGESVTVTR2+	844.9463	2	0	32	29	150-1700
MCM3>GGYTSGTFR#2+	478.229	2	0	32	25	150-1000
MCM3>GGYTSGTFR2+	473.2249	2	0	32	25	150-900
MCM3>LIVNVNDLR#2+	533.3182	2	0	32	25.5	150-1100
MCM3>LIVNVNDLR2+	528.314	2	0	32	25.5	150-1100
MCM3>SVDVILDDDLVDK#2+	727.3792	2	0	32	25.5	150-1500
MCM3>SVDVILDDDLVDK2+	723.3721	2	0	32	25.5	150-1400
S100P>ELPGFLQSGK#2+	542.2999	2	0	32	27	150-1100
S100P>ELPGFLQSGK2+	538.2928	2	0	32	27	150-1100
S100P>YSGSEGSTQTLTK#2+	683.8324	2	0	32	28	150-1400
S100P>YSGSEGSTQTLTK2+	679.8253	2	0	32	28	150-1400
